# A comparative study of identical VMAT about two adjacent targets with and without fixed-jaw technique

**DOI:** 10.1186/s13014-019-1284-2

**Published:** 2019-05-08

**Authors:** Kai Xie, Hongfei Sun, Liugang Gao, Tao Lin, Jianfeng Sui, Xinye Ni

**Affiliations:** 1 Radiotherapy Department, Second People’s Hospital of Changzhou, Nanjing Medical University, Changzhou, 213003 People’s Republic of China; 2 Center for Medical Physics, Nanjing Medical University, Changzhou, 213003 People’s Republic of China

**Keywords:** VMAT, Adjacent targets, MLC transmission, Fixed-jaw, Dosimetry

## Abstract

**Background:**

The radiation transmission through the multileaf collimators is undesired in modern techniques such as volumetric modulated arc therapy (VMAT). According to identical plans, in this study, we aim to investigate the dosimetric impact of jaw tracking on the VMAT plans on two adjacent targets.

**Methods:**

Two treatment plans were designed for eight pelvic (cervical) patients with two targets using the same optimization parameters. The original plan (O-plan) used automatically selected jaw positions. In the new plan (F-plan), the jaws were fixed to block two targets in two beams. The dosimetric parameters of the two plans were compared to evaluate the improvement of dose sparing for the body volume between two targets (named interOAR) in F-VMAT.

**Results:**

The mean dose of interOAR reduced significantly from 654.96 ± 113.38 cGy for O-VMAT, to 490.84 ± 80.26 cGy for F-VMAT (*p* = 0.018). The monitor units (MUs) in the F-plans were 1.49-fold higher than that in the O-plan. The F and O-plan performed similarly in target dose homogeneity. The differences in Dmax of spinal cord, Dmax of spinal cord planning organ at risk volume, and V20, V30, and V40 of the intestine were insignificant.

**Conclusions:**

VMAT plans with the fixed-jaw method can reduce the volume between two targets effectively. However, despite the plan quality, the method can only be used when the regular methods cannot reach the clinical requirements for critical organs because of additional MUs.

## Background

A multileaf collimator (MLC) is essential to realize the intensity distributions required in intensity-modulated radiotherapy (IMRT) [1, 2] and volumetric-modulated arc radiotherapy (VMAT) [3–6]. For only covered by MLC, the transmitted dose rate can be 0.90–4.40% (6 MV photon) higher than that shielded by both MLC and jaws. Without protection from jaws, the critical organs may receive doses from MLC leaf transmission and leakage. If any of the organs (such as lens, ovaries, and testicles) is extremely sensitive to low tolerance dose, then its received dose may be higher than the dosage it can tolerate.

Generally, multiple lesions are common in radiotherapy. For targets far from each other, the planning design is carried out separately for each target; for targets close to one another, the same plan is generally used for simultaneous optimization [7–10]. Then, the design of the treatment plans is the key to radiotherapy and the focus of our study.

The benefits of jaw tracking have been assessed for IMRT on the basis of the same plan except for the jaw settings [11–13]. However, whether or not the radiation dose of the critical organ between targets can be reduced by fixing jaw position appropriately in VMAT has not been verified. In this work, a fixed-jaw method on VMAT plans was developed to protect the body volume between two targets and evaluated for the planning target volume (PTV) coverage and organ at risk (OAR) protection of eight pelvic (cervical) cancer patients.

## Materials and methods

### Patients

Eight pelvic (cervical) patients with two targets from June to December 2017 in our hospital were selected. The mean age of these patients was 56 ± 7 years old, and the median age was 58 years old. The continuous CT scan images with 5 mm thickness were transferred to the Monaco 5.1 treatment system (Elekta AB, Stockholm, Sweden). The target volumes and OARs were contoured by an experienced physician, where the superior side of PTV was PTV1, the inferior side was PTV2; PTV1 and PTV2 were combined into PTV. To evaluate the low dose transmission further, we contoured the body volume between PTV1 and PTV2 as interOAR. The mean volumes of PTV1 and of all patients were 300.04 and 489.38 cm^3^, respectively. A total dose of 45 Gy in 25 fractions were prescribed to cover 95% of the PTV volume.

The Elekta Infinity linear accelerator (Elekta AB) equipped with 80 pairs of MLC with a thickness of 5 mm was used in this study [14]. The TPS was the Monaco system, and Monte Carlo algorithm served as the algorithm. The computational grid was 3 mm, with 1% computational accuracy.

### VMAT plans

In the Monaco planning system, a commonly used coplanar plan with Gantry being 0° and couch being 0° was selected, and the energy was 6 MV. For each patient, two plans were designed, as follows:

O-VMAT: one beam and two arcs; the isocenter was the center of PTV. O-VMAT used automatically selected jaw positions; the gantry rotated from − 180° to 180°clockwise and then rotated to − 180° anticlockwise.

F-VMAT: two beams and two arcs; the isocenter was the PTV center. This plan used fixed-jaw technology, where the jaws in beam 1 and 2 were fixed to block PTV1 and PTV2, respectively. Beam 1 rotated from − 180° to 180° clockwise, and beam 2 rotated from 180° to − 180° anticlockwise.

### Statistics

The monitor units (MUs), Paddick conformity index (CI) [15] and homogeneity index (HI) [16] were used to compare the differences among different VMAT planning results. The Paddick CI was defined as CI = (TVPV)^2^/(TV × PV), where PV is the volume contained by the prescriptive dose, TVPV is the target volume contained by prescriptive dose, and TV is the target volume. HI evaluates the dose homogeneity to the target volume, and is defined as HI = (D5%) / (D95%), where D5% and D95% are minimum doses delivered to 5 and 95% of the target volume, respectively. A HI of 1 signifies that the absorbed dose distribution is nearly homogeneous. Dmax and Dmean are the maximum and average doses delivered to the OARs, respectively. V_n Gy_ (%) is the percentage of the organ volume receiving ≥ n Gy. The following parameters were assessed: V_20 Gy_, V_30 Gy_, and V_40 Gy_ of the intestine, Dmax of spinal cord; Dmax of spinal cord planning OAR volume (PRV); and Dmean, V_20 Gy_, V_30 Gy_, and V_40 Gy_ of the interOAR. The mean values and standard deviation were collected, and the results were compared by a non-parametric Wilcoxon signed-rank test. All computations at *p* = 0.05 level for statistical significance, were performed using SPSS version 20.0 (IBM, Chicago, IL).

## Results

Table 1 summarizes the results of the plan quality metrics for both O-VMAT and F-VMAT. The results from hypothesis testing are shown as follows. While Dmean of interOAR reduced significantly from 654.96 ± 113.38 cGy for O-VMAT to 490.84 ± 80.26 cGy for F-VMAT (*p* = 0.018), the MU value significantly increased from 766.40 ± 97.46 for O-VMAT to 1133.21 ± 162.11 for F-VMAT (*p* = 0.012). Given that the main doses of interOAR were from MLC transmission, the low component such as V3, V5 and V10 also decreased significantly (*p* = 0.018, 0.018, 0.018).

**Table 1:** Comparison of PV and OAR doses between O-VMAT and F-VMAT

Regions of interest	O-VMAT	F-VMAT	*p* Values
PTV			
CI	0.831 ± 0.093	0.830 ± 0.090	0.833
HI	1.055 ± 0.014	1.050 ± 0.011	0.157
Spinal cord			
Dmax (cGy)	3625.19 ± 376.55	3575.04 ± 343.55	0.208
Spinal cord PRV			
Dmax (cGy)	4148.33 ± 280.23	4124.20 ± 289.31	0.263
Intestine			
V_20_ (%)	29.68 ± 12.49	30.15 ± 11.89	0.624
V_30_ (%)	16.68 ± 11.66	16.07 ± 11.65	0.161
V_40_ (%)	9.44 ± 9.10	9.72 ± 9.41	0.123
interOAR^a^			
Dmean (cGy)	654.96 ± 113.38	490.84 ± 80.26	0.018
V_3_ (%)	70.11 ± 17.86	40.17 ± 12.91	0.018
V_5_ (%)	37.40 ± 12.86	26.71 ± 7.03	0.018
V_10_ (%)	17.47 ± 4.56	13.25 ± 2.26	0.018
MUs	766.40 ± 97.46	1133.21 ± 162.11	0.012

^a^: except for the first case because it was an outlier in the box plot

For PTV, the difference between CI of O-VMAT, (0.831 ± 0.093), and F_VMAT, (0.830 ± 0.090) was insignificant (*p* = 0.833), which was the same as HI (*p* = 0.157). Consistently, the differences in Dmax of spinal cord, Dmax of spinal cord PRV, and V20, V30, V40 of intestine were insignificant.

**Fig. 1:**
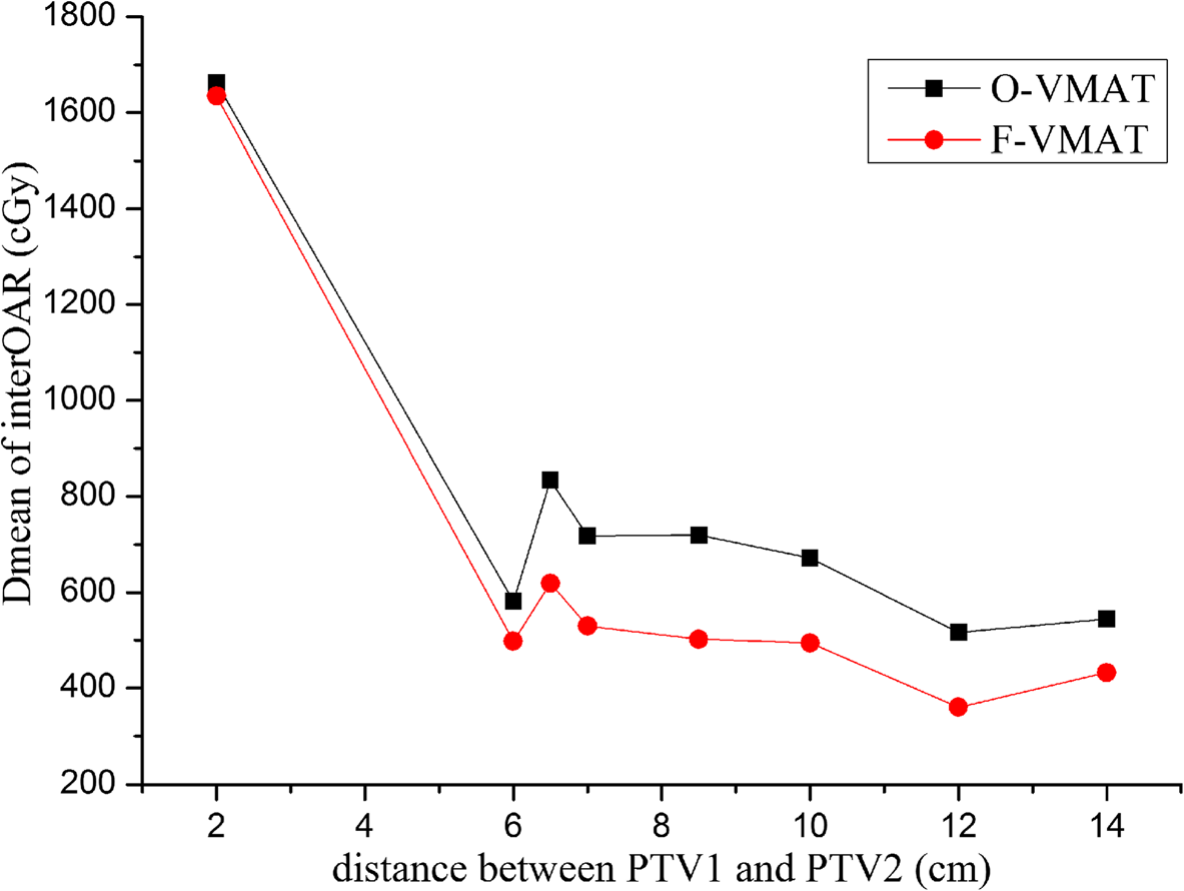
Relationship among Dmean of interOAR and distance between PTV1 and PTV2

Figure 1 showed the effect of distance between PTV1 and PTV2 on Dmean of interOAR. The first case was an outlier in the box plot and was not in interOAR calculation. This figure revealed that except for exactly small distance, Dmean of interOAR can maintain a value of approximately 650 cGy in O-VMAT plans and 490 cGy in F-VMAT.

**Fig. 2:**
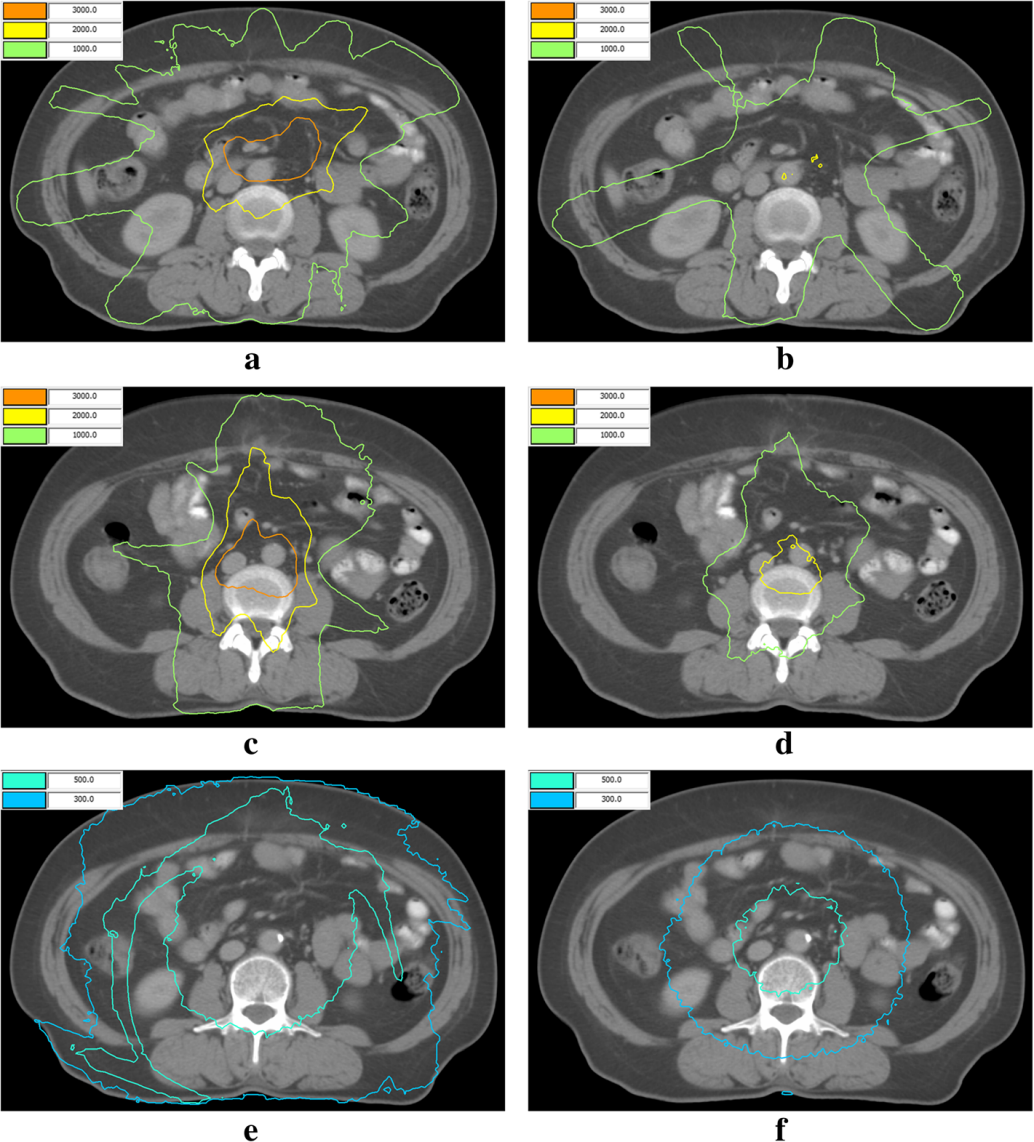
Dose distribution of interOAR in different places from one patient. **a** 10 mm from PTV1 in O-VMAT; (**b**) 10 mm from PTV1 in F-VMAT; (**c**) 10 mm from PTV2 in O-VMAT; (**d**) 10 mm from PTV2 in F-VMAT; (**e**) at the center of interOAR in O-VMAT, and (**f**) at the center of interOAR in F-VMAT

Given the steep dose gradients in the jaws margin, the doses of interOAR near PTV1 and PTV1 in F-VMAT were smaller than that in O-VMAT. Figure 2 showed the dose distribution of interOAR volume in 10 mm from PTV1 and PTV2, and in the interOAR center.

## Discussion

This study assessed a method of jaw positioning during VMAT to protect the OARs between two adjacent targets further. To evaluate the improvements associated with the fixed-jaw technique, we introduced and compared F-VMAT with the O-VMAT. In O-VMAT, the jaw positions were automatically set to cover PTV. In F-VMAT, the jaw positions were set to cover PTV1 and PTV2 in the two beams, respectively.

A relatively decrease dose in interOAR was observed in F-VMAT, where MU increased nearly half compared with O-VMAT. The differences were insignificant in other metrics. This result was similar to the conclusion drawn by Clark et al. [17]

In Monaco, when the jaws were set to cover PTV, the margin was set to 10 mm as default. The distance between the two targets in the first case was 2 cm, which was only twofold higher than the margin. Thus, the fixed-jaw method did not reduce the interOAR dose. The dose gradient also helped make the interOAR dose in case 1 higher than the others. The interOAR dose in case 2 was smaller than the others because PTV2 in case 2 was near the skin. InterOAR was defined as the entire body volume between two targets that may result in no correlation between interOAR dose and targets volume.

Feng et al. [18] showed that when the jaw tracking was applied, the mean doses were significantly lower than those when using static jaw technique. Chen et al. [19] reported that the patients’s pelvic radiation dosage can be effectively reduced by using the fixed-jaw method, compared with the routine jaw auto-selected method. Wu et al. [20] showed that in patients with head and neck, thoracic, abdominal, and pelvic cancer, OAR irradiation can be reduced by locking the jaw positions in the VMAT plans. These studies showed that fixed-jaw method can improve the protective effect of OAR. Our study applied this method to patients with two adjacent targets, and this method can acquire a similar effect, particularly improved interOAR protection.

As a limitation of cases number, interOAR did not contain critical organs (such as lens, ovaries, and testicles). Additional cases with different distance between two targets and various volumes of PTV should be collected.

## Conclusions

For the two adjacent targets, the VMAT plans using fixed-jaw to cover PTV1 and PTV2 in two beams performed better than normal VMAT plans in terms of interOAR dose, while the other OAR metrics remained the same. Although significant dosimetric benefits were found, the MUs will increase when the fixed-jaw method is used. Despite its better performance, this method should only be used to protect critical organs (such as lens, ovaries, and testicles) when the regular methods cannot reach the clinical requirements because of more MUs.

## References

[1] Hussein M, South CP, Barry MA, Adams EJ, Jordan TJ, Stewart AJ (2016). Clinical validation and benchmarking of knowledge-based IMRT and VMAT treatment planning in pelvic anatomy. Radiother Oncol.

[2] Low DA, Moran JM, Dempsey JF, Dong L, Oldham M (2011). Dosimetry tools and techniques for IMRT. Med Phys.

[3] Rao M, Yang W (2010). Comparison of Elekta VMAT with helical tomotherapy and fixed field IMRT: plan quality, delivery efficiency and accuracy. Med Phys.

[4] Verbakel WFAR, Cuijpers JP, Hoffmans D, Bieker M, Slotman BJ, Senan S (2009). Volumetric intensity-modulated arc therapy vs. conventional IMRT in head-and-neck Cancer: a comparative planning and Dosimetric study. Int J Radiat Oncol Biol Phys.

[5] Lee TF, Chao PJ, Ting HM, Lo SH, Wang YW, Tuan CC (2011). Comparative analysis of SmartArc-based dual arc volumetric-modulated arc radiotherapy (VMAT) versus intensity-modulated radiotherapy (IMRT) for nasopharyngeal carcinoma. J Appl Clin Med Phys.

[6] Holt A, Van Gestel D, Arends MP, Korevaar EE, Schuring D, Kunzebusch MM (2013). Multi-institutional comparison of volumetric modulated arc therapy vs. intensity-modulated radiation therapy for head-and-neck cancer: a planning study. Radiat Oncol.

[7] Roberge D, Ruo R, Souhami L (2006). Killing two birds with one stone : a dosimetric study of dual target radiosurgery using a single isocenter. Technol Cancer Res Treat.

[8] Clark GM, Popple R, Prendergast BM, Spencer SA, Thomas EM, Stewart JG (2012). Plan quality and treatment planning technique for single isocenter cranial radiosurgery with volumetric modulated arc therapy. Pract Radiat Oncol.

[9] Iwai Y., Ozawa S., Ageishi T., Pellegrini R., Yoda K. (2014). Feasibility of single-isocenter, multi-arc non-coplanar volumetric modulated arc therapy for multiple brain tumors using a linear accelerator with a 160-leaf multileaf collimator: a phantom study. Journal of Radiation Research.

[10] Mayo C, Ding L, Addesa A, Kadish SP, Fitzgerald TJ, Moser RP (2010). Initial experience with volumetric IMRT (RapidArc) for intracranial stereotactic radiosurgery. Int J Radiat Oncol Biol Phys.

[11] Kim J, Kim SP, Choe B, Suh T, Park S, Jo S (2015). Clinical assessment of the jaw-tracking function in IMRT for a brain tumor. J Korean Phys Soc.

[12] Zhang W, Lu J, Chen J, Zhai T, Huang B, Li D (2016). A Dosimetric study of using fixed-jaw volumetric modulated arc therapy for the treatment of nasopharyngeal carcinoma with cervical lymph node metastasis. PLoS One.

[13] Sarah J, George S, Stephen K, Mohammed S, Allen WR, Lin SH (2012). Dosimetric effects of jaw tracking in step-and-shoot intensity-modulated radiation therapy. J Appl Clin Med Phys..

[14] Nevelsky A, Ieumwananonthachai N, Kaidarperson O, Barderoma R, Nasrallah H, Benyosef R (2013). Hippocampal-sparing whole-brain radiotherapy using Elekta equipment. J Appl Clin Med Phys..

[15] Paddick I (2000). A simple scoring ratio to index the conformity of radiosurgical treatment plans. Tech Note J Neurosurg.

[16] Semenenko VA, Reitz B, Day E, Qi XS, Miften M, Li XA (2008). Evaluation of a commercial biologically based IMRT treatment planning system. Med Phys.

[17] Clark GM, Popple R, Young PE, Fiveash JB (2010). Feasibility of single-Isocenter volumetric modulated arc radiosurgery for treatment of multiple brain metastases. Int J Radiat Oncol Biol Phys.

[18] Feng Z, Wu H, Zhang Y, Zhang Y, Cheng J, Su X (2015). Dosimetric comparison between jaw tracking and static jaw techniques in intensity-modulated radiotherapy. Radiat Oncol.

[19] Chen J, Chen X, Huang M, Dai J (2014). A fixed-jaw method to protect critical organs during intensity-modulated radiotherapy. Med Dosim.

[20] Wu H, Jiang F, Yue H, Hu Q, Zhang J, Liu Z (2016). A comparative study of identical VMAT plans with and without jaw tracking technique. J Appl Clin Med Phys..

